# High prevalence of vector-borne pathogens in the blood of clinically healthy dogs in Hong Kong

**DOI:** 10.1186/s13071-025-06853-5

**Published:** 2025-07-20

**Authors:** Thamali Manathunga, Mariaelisa Carbonara, Omid Nekouei, Jairo Alfonso Mendoza-Roldan, Wing Yan Jacqueline Tam, Frederic Beugnet, Domenico Otranto, Vanessa R. Barrs

**Affiliations:** 1 Department of Veterinary Clinical Sciences, Jockey Club College of Veterinary Medicine and Life Sciences, City University of Hong Kong, Kowloon Tong, Hong Kong; 2 Department of Veterinary Medicine, University of Bari, Bari, Italy; 3 Department of Infectious Diseases and Public Health, City University of Hong Kong, Kowloon Tong, Hong Kong; 4 Boehringer-Ingelheim Animal Health, Lyon, France; 5 Centre for Animal Health and Welfare, City University of Hong Kong, Kowloon Tong, Hong Kong

**Keywords:** *Anaplasma* spp., *Babesia gibsoni*, Canine vector-borne diseases, *Dirofilaria* spp., *Dirofilaria asiatica*, *Ehrlichia canis*, *Hepatozoon canis*, Hong Kong, *Leishmania* spp., *Trypanosoma evansi*

## Abstract

**Background:**

Leishmaniosis and other canine vector-borne diseases (CVBDs) pose a major risk for veterinary and public health globally, especially where humans and dogs live in close proximity. Although mosquito and tick vectors are abundant in Hong Kong, surveillance for CVBDs has been limited.

**Methods:**

A serological and molecular survey of 158 healthy owned (*n* = 64) and free-roaming unowned (*n* = 94) dogs with outdoor access in Hong Kong was performed to determine CVBD prevalence. Point-of-care (POC) immunoassays were used to detect (i) antibodies to *Leishmania* spp., *Ehrlichia* spp., and *Anaplasma* spp., and (ii) *Dirofilaria immitis* and *Angiostrongylus vasorum* antigens, in canine sera. Conventional polymerase chain reaction (PCR) was also carried out to detect the molecular prevalence of all five pathogens as well as *Hepatazoon canis*,* Babesia gibsoni*, and *Trypanosoma evansi*. In addition, for *Leishmania* spp. detection, an immunofluorescence antibody test (IFAT) was performed on all serum samples, followed by real-time PCR of seropositive samples to detect *Leishmania* spp. DNA. The agreement between tests was assessed by Cohen’s kappa statistic, and logistic regression analysis was applied to identify potential risk factors.

**Results:**

Overall, 45.6% of dogs tested positive on molecular and/or serological tests for at least one pathogen, with the highest prevalence recorded for *Dirofilaria* spp. (20.9%), followed by *B. gibsoni* (15.2%), *Leishmania* spp. (11.4%), *Anaplasma* spp. (7.6%), *H. canis* (4.4%), *Ehrlichia* spp. (3.8%), and *A. vasorum* (0.6%). No *T. evansi* DNA was detected. Co-infections or co-pathogen exposure occurred in 16.5% of samples. Of the 33 *Dirofilaria* spp.-positive dogs, two were identified by sequencing as *Dirofilaria asiatica*, and the remaining 31 were *D. immitis*. No significant risk factors for infection or exposure were identified.

**Conclusions:**

This is the first epidemiological survey of *Leishmania* spp. infection in dogs from Hong Kong, highlighting the need for surveillance of competent vectors and further investigation of disease status in dog populations to confirm whether this pathogen is endemic. Given the high prevalence of CVBD, especially of *D. immitis,* preventive and control measures are advocated in order to mitigate risks to canine health and zoonotic infection.

**Graphical abstract:**

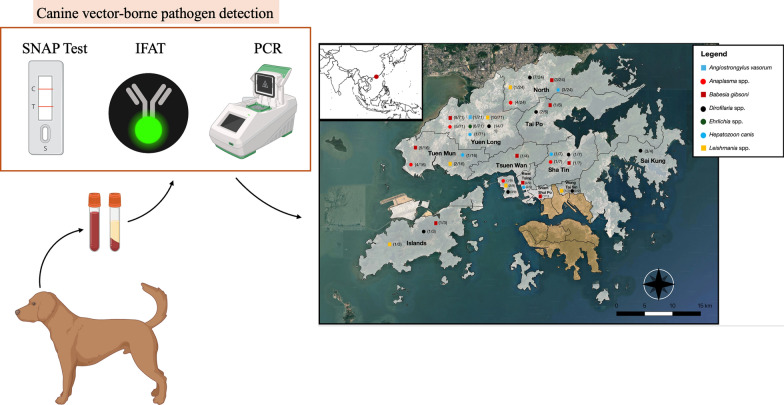

**Supplementary Information:**

The online version contains supplementary material available at 10.1186/s13071-025-06853-5.

## Background

A plethora of biotic (e.g., the introduction of arthropod vectors, dog movements, closer proximity of wildlife populations due to habit loss) and abiotic factors (e.g., global warming and urbanization of rural areas) may represent a risk for increasing the abundance and geographical distribution of canine vector-borne diseases (CVBDs), including those of zoonotic concern [1]. Although the Hong Kong Special Administrative Region (SAR), located at the southeastern tip of China, is a densely populated cosmopolitan city of 7.4 million people [2], the proportion of dog ownership is relatively low compared to many other countries, with only 5.7% of total households owning at least one dog [3].

Free-roaming “village” dogs represent the most common carnivores in Hong Kong SAR and pose a risk for the transmission of CVBDs, many of which are zoonotic [4]. Indeed, Hong Kong has a subtropical monsoon climate, providing favorable environmental conditions for vector proliferation. For example, *Aedes albopictus* mosquitoes, the primary vectors of canine filariosis, are highly abundant in Hong Kong [5], with *Dirofilaria immitis* causing canine heartworm disease [6]. This filarioid is the agent of human dirofilariasis worldwide, along with other species such as *Dirofilaria repens* and *Dirofilaria asiatica* (formerly *Dirofilaria* sp. “hongkongensis”), the latter causing subcutaneous nodules in cats and dogs [7] as well as in humans [8, 9].

Despite limited tick surveillance, four genera (i.e., *Rhipicephalus*, *Haemaphysalis*, *Ixodes*, and *Hyalomma*) have been recorded as being present in Hong Kong by the Food and Environmental Hygiene Department (FEHD) [10]. In particular, *Rhipicephalus sanguineus* sensu lato (s.l.) and *Haemaphysalis longicornis* are adapted to the subtropical climate of Hong Kong and may transmit *Babesia vogeli*, *Ehrlichia canis* (vectored by *R. sanguineus* s.l.), and *Babesia gibsoni* (vectored by *H. longicornis*) [11, 12].

From the limited investigations that have been performed so far, babesiosis, due to *B. gibsoni*, is the most common CVBD in Hong Kong [6, 13, 14], with a molecular prevalence ranging from 3.8% to 31% in owned dogs, and of up to 44% in free-roaming unowned dogs [6, 13, 14]. By contrast, *B. vogeli* has seldom been reported in dogs from Hong Kong (molecular prevalence < 5%) [13, 14]. *Babesia hongkongensis* has been detected at a low prevalence (1.7%) in free-roaming cats but not dogs from Hong Kong [15, 16]. Other vector-borne pathogens (VBPs), including *E. canis*, *Hepatozoon canis*, *Anaplasma platys*, and the potentially zoonotic *Anaplasma phagocytophilum*, have also been reported with low occurrence among dogs from Hong Kong [6, 13, 14].

So far, canine leishmaniosis has only been reported in two purebred dogs (Belgian Malinois) living in the same household in Hong Kong [17]. The first case was a dog imported from the USA, which presented with chronic cutaneous leishmaniosis, while the second case was autochthonous, presenting with systemic leishmaniosis [17]. Both cases were due to *Leishmania infantum*, the most important zoonotic *Leishmania* species, causing cutaneous and/or visceral signs in dogs and humans, depending on the host’s immune response. However, the primary vector (i.e., phlebotomine sand flies) has not yet been reported in Hong Kong, and non-vectorial, transplacental, or transmission by direct contact was suspected in both cases [17].

Given the paucity of information about CVBDs in this region of China, this study aimed to determine the prevalence of leishmaniosis and other CVBDs in healthy dogs with outdoor access in Hong Kong, using a comprehensive range of molecular and serological tests.

## Methods

### Sample collection

**Fig. 1:**
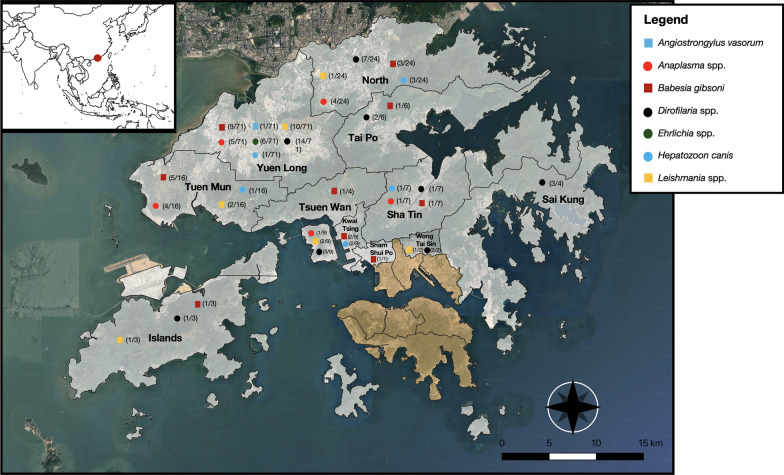
Map of the study areas indicated by district, showing the location of dogs enrolled, according to their positivity to vector-borne pathogens. Tan shaded regions indicate locations where only negative samples were detected, while gray-shaded regions represent areas where positive samples were identified

From December 2022 to March 2023, blood samples were collected from 158 dogs presented to a neutering clinic in the New Territories of Hong Kong (Fig. 1). All dogs were assessed by the attending veterinarians as healthy. Age, sex, breed, neuter status, outdoor walks per day, outdoor environment, and primary residential district were recorded for each dog. Whole blood was collected into ethylenediaminetetraacetic acid (EDTA) and plain tubes. The serum was separated after centrifugation at 10,000×*g* for 10 min. Sera and EDTA blood samples were stored at – 80 ºC.

### Serological antibody and antigen tests

An immunofluorescence antibody test (IFAT) was used to detect anti-*Leishmania* immunoglobulin G (IgG) antibodies, as reported previously [18]. Samples that showed clear promastigote fluorescence at a cut-off dilution of 1:80 were considered positive and then titrated until no fluorescence was observed. *Leishmania* antibodies were also detected using a commercially available rapid enzyme-linked immunosorbent assay (ELISA)-based test kit (SNAP^®^ Leish 4Dx^®^ test; IDEXX Laboratories, Westbrook, ME, USA).

Serum samples were tested for *D. immitis* antigen using two point-of-care (POC) immunoassays (SNAP^®^ Heartworm RT test kit and SNAP^®^ Leish 4Dx^®^ test; IDEXX Laboratories, Westbrook, ME, USA). *Angiostrongylus vasorum* antigen was detected using a commercial rapid immunochromatographic test kit (Angio Detect™ Test, IDEXX Laboratories, Westbrook, ME, USA). Antibodies against *Anaplasma* spp. (i.e., *A. phagocytophilum*, *A. platys*) and *Ehrlichia* spp. (i.e., *E. canis*, *E. ewingii*, *E. chaffeensis*) were detected using the SNAP^®^ Leish 4Dx^®^ test (IDEXX Laboratories, Westbrook, ME, USA). The SNAP^®^ Leish 4Dx^®^ test has been validated for use in dogs [19], as has the Angio Detect™ test [20] and the SNAP^®^ Heartworm RT test [21].

### Molecular analysis

Genomic DNA was extracted from EDTA blood using the QIAamp DNA Blood and Tissue Kit (Qiagen GmbH, Hilden, Germany), according to the manufacturer’s instructions. For all samples, DNA of flagellated protozoa (i.e., *Leishmania* spp., *Trypanosoma evansi*), Rickettsiales (*Anaplasma* spp., *Ehrlichia* spp., and *Wolbachia* spp.), tick-borne apicomplexan protozoa (*Babesia* spp. and *Hepatozoon* spp.), and nematodes (i.e., *Dirofilaria* spp., *A. vasorum*) was searched for using conventional polymerase chain reaction (PCR) protocols listed in Table 1. All PCR products were analyzed by electrophoresis on 2% agarose gels, purified, and subjected to Sanger sequencing. Sequences were edited using Geneious Prime^®^ 2024.0.3, and the Basic Local Alignment Search Tool (BLAST) on the National Center for Biotechnology Information (NCBI) GenBank was used to determine species identity.

**Table 1:** Pathogen targeted in blood samples, and list of primers used in the conventional PCR protocols

Pathogen	Target gene	Primer	Amplicon length (base pairs, bp)	References
*Leishmania* spp.	Kinetoplast DNA (kDNA) minicircle	MC1 (5′ GTTAGCCGATGGTGGTCTTG 3′) MC2 (5′ CACCCATTTTTCCGATTTTG 3′)	447	[64]
*Dirofilaria* spp.	Cytochrome c oxidase subunit-1 (*cox*1)	Diro_COX1_F (5′ GCTTTGTCTTTTTGGTTTACTTTT 3′) Diro_COX1_R (5′ TCAAACCTCCAATAGTAAAAAGAA 3′)	880	[65]
*Angiostrongylus vasorum*	*18S* rRNA	NC18SF1 (5′ AAAGATTAAGCCATGCA 3′) NC5BR (5′ GCAGGTTCACCTACAGAT 3′)	1700	[66]
*Anaplasma* spp./*Ehrlichia* spp./*Wolbachia pipientis*^a^	*16S* rRNA	EHR-16SD (5′ GGTACCYACAGAAGAAGTCC 3′) EHR-16SR (5′TAGCACTCATCGTTTACAGC 3′)	345	[67]
*Babesia* spp./*Hepatozoon* spp./	*18S* rRNA	HepF300 (5′ GTTTCTGACCTATCAGCTTTCGACG 3′) HepR900 (5′ CAAATCTAAGAATTTCACCTCTGAC 3′)	581	[68]
*Trypanosoma evansi*	Variant surface glycoprotein gene (*vsg* gene)	TevF (5′ TGCAGACGACCTGACGCTACT 3′) TevR (5′ CTCCTAGAAGCTTCGGTGTCCT 3′)	227	[69]

^a^Endosymbiont of filarioids

For samples that were seropositive for *Leishmania* spp. antibodies by IFAT and/or commercial test kit, a real-time PCR (qPCR) was performed to detect *L. infantum* kinetoplast DNA (kDNA) minicircle fragments. A duplex real-time PCR assay (dqPCR) was also performed to detect both *L. infantum* and *Leishmania tarentolae* DNA. Details of the primers, probes, and protocols used for qPCR assays are provided in Table 2.

**Table 2:** Primers and probes used for quantitative polymerase chain reaction (qPCR) of *Leishmania*-seropositive samples

qPCR target	Primers/probes	Amplicon length (base pairs, bp)	Reference
Kinetoplast minicircle DNA from *L. infantum*	LEISH-1 (5′ AACTTTTCTGGTCCTCCGGGTAG 3′) LEISH-2 (5′ ACCCCCAGTTTCCCGCC 3′) TaqMan-minor groove binder probe: (FAM-5′AAAAATGGGTGCAGAAAT 3′ non-fluorescent quencher-MGB)	120	[70]
Duplex PCR for detection of *L. infantum* and *L. tarentolae* internal transcribed spacer 1 (ITS1) DNA	ITS1-F (5′ GCAGTAAAAAAAAGGCCG 3′) ITS1-R (5′ CGGCTCACATAACGTGTCGCG 3′)	150	[71]

### Data analysis

To calculate the overall prevalence of *Dirofilaria* spp., *A. vasorum*, *Ehrlichia* spp., *Anaplasma* spp., and *Leishmania* spp., a sample was considered positive when it tested positive on at least one serological assay and/or by PCR. The overall prevalence of *Babesia* spp. and *Hepatozoon* spp. was calculated based only on molecular results.

To measure the level of agreement between different tests for each pathogen, Cohen’s kappa statistics were calculated [22]. The level of agreement was defined as follows: no agreement (κ = 0), slight agreement (κ = 0.01–0.2), fair agreement (κ = 0.21–0.4), moderate agreement (κ = 0.41–0.6), substantial agreement (κ = 0.61–0.8), or almost perfect agreement (κ = 0.81–1). The potential associations between exposure to tick-borne pathogens and independent variables (e.g., sex, age, environment, being stray, and outdoor walk frequency) were evaluated using simple logistic regressions with a 0.05 significance level. All statistical analyses were conducted in Stata v18 (StataCorp LLC, College Station, TX, USA).

## Results

Of the 158 dogs included in this study, 94 (59.5%) were free-roaming and unowned, while 64 (40.5%) were owned. All dogs were mongrels and had outdoor access. There were 53 sexually intact male dogs (33.5%) and 105 intact female dogs (66.5%), ranging in age from 5 months to 6.5 years (median 1.4 years). One hundred dogs (63.3%) were older than 1 year.

Overall, 72 (45.6%; 95% confidence interval [CI] 37.8–53.3) dogs tested positive on molecular and/or serological tests for at least one pathogen (Fig. 1), with the highest prevalence recorded for *Dirofilaria* spp. (20.9%; 95% CI 14.6–27.2). Other pathogens detected in decreasing order of prevalence were *B. gibsoni* (15.2%; 95% CI 9.6–20.8), *Leishmania* spp. (11.4%; 95% CI 6.4–16.4), *Anaplasma* spp. (7.6%; 95% CI 3.5–11.7), *H. canis* (4.4%; 95% CI 1.2–7.6), *Ehrlichia* spp. (3.8%; 95% CI 0.8–6.8), and *A. vasorum* (0.63%; 95% CI 0.0–3.5) (Table 3). No *T. evansi* DNA was detected.

**Table 3:** Serological and molecular results of canine vector-borne pathogens detected in 158 dogs from Hong Kong

Pathogen	Positive Ag or Ab tests No. (%; 95% CI)	PCR-positive No. (%; 95% CI)	Species identified by sequencing (no.)	Ag- or Ab-positive/PCR-positive	Ag- or Ab-positive/PCR-negative	Ag- or Ab-negative/PCR-positive	Total prevalence: no. of positive on Ag, Ab, and/or molecular tests (%; 95% CI)
*Dirofilaria* spp.	30 (19; 12.9–25.1)	19 (12; 7.0–17.1)	*Dirofilaria immitis* (16) *Dirofilaria asiatica* (2) *Wolbachia pipientis* (15)	16	14	3	33 (20.9, 14.6–27.2)
*Angiostrongylus vasorum*	1 (0.6; 0.02–3.5)	0 (0)	N/A	N/A	N/A	N/A	1 (0.6; 0–3.5)
*Anaplasma* spp.	12 (7.6; 3.5–11.7)	4 (2.5; 0.1–5.0)	*Anaplasma platys* (4)	4	8	0	12 (7.6; 3.5–11.7)
*Leishmania* spp.	18 (11.4; 6.4–16.4)	0 (0)	N/A	N/A	18	N/A	18 (11.4; 6.4–16.4)
*Ehrlichia* spp.	5 (3.2; 0.4–5.9)	3 (1.9; 0.4–5.5)	*Ehrlichia canis*	2	3	1	6 (3.8; 0.8–6.8)
*Babesia* spp.	N/A	24 (15.2; 9.6- 20.8)	*Babesia gibsoni* (24)	N/A	N/A	N/A	24 (15.2; 9.6–20.8)
*Hepatozoon* spp.	N/A	7 (4.4; 1.2–7.6)	*Hepatozoon canis* (7)	N/A	N/A	N/A	7 (4.4; 1.2–7.6)
*Trypanosoma evansi*	N/A	0 (0)	N/A	N/A	N/A	N/A	0 (0)

*Ab* antibody, *Ag* antigen, *CI* confidence interval, *N/A* not applicable

Of the 18 (11.4%; 95% CI 6.4–16.4) dogs seropositive for *Leishmania* spp., 17 were positive on IFAT alone and one on the POC immunoassay. IFAT titers ranged from 1:80 to 1:320, including 10 dogs with a titer of 1:80, five with a titer of 1:160, and two with a titer of 1:320. *Leishmania* spp. DNA was not detected in any blood sample.

Among the 33 (20.9%; 95% CI 14.6–27.2) samples positive for *Dirofilaria* spp., 19 tested positive on the *cox*1 gene PCR for *Dirofilaria* spp. and/or had *16S* ribosomal RNA (rRNA) *Wolbachia* sequences with 100% nucleotide identity to *Wolbachia pipientis* (GenBank Accession no: OR939250.1). Cohen’s kappa statistic, κ = 0.83, indicated perfect agreement between *Dirofilaria* spp. results by *cox*1 PCR and the *Wolbachia* results by *16S* rRNA PCR. Similarly, both SNAP^®^ Leish 4Dx^®^ test (IDEXX Laboratories, Westbrook, ME, USA) and SNAP^®^ Heartworm RT test kit (IDEXX Laboratories, Westbrook, ME, USA) had almost perfect agreement in detecting *D. immitis* antigens (κ = 0.84). No agreement between IFAT and SNAP^®^ Leish 4Dx^®^ test (IDEXX Laboratories, Westbrook, ME, USA) was observed for *Leishmania* antibody detection (κ = 0). Discordant results were observed between molecular and serological tests for *Dirofilaria*, *Ehrlichia*, and *Anaplasma* (Table 3). In particular, two samples identified molecularly as *D. asiatica* showed different results, with one testing antigen-positive on SNAP^®^ Heartworm RT test kit (IDEXX Laboratories, Westbrook, ME, USA) and the other negative. The *W. pipientis* sequence was also amplified from the *16S* rRNA PCR assay of the antigen-positive *D. asiatica* sample.

Based on both serological and molecular results, 16.5% (26/158; 95% CI 10.7–22.2) of dogs were infected or exposed to more than one VBP (Table 4). Among the *Leishmania*-seropositive samples, 88.9% (16/18; 95% CI 65.3–98.6) had co-infections or were exposed to at least one other pathogen. In addition, 29.1% (46/158; 95% CI 22.0–36.2) of dogs tested positive by serology or PCR for at least one tick-borne pathogen. Three dogs were co-infected with more than one tick-borne pathogen (Table 4).

**Table 4:** Number of dogs with co-pathogen infections or exposure based on all test results (serological and molecular)

Pathogen combinations	No. co-infected cases
*Dirofilaria* spp., *Leishmania* spp.	2
*Dirofilaria* spp., *Anaplasma* spp.	1
*Dirofilaria* spp., *Babesia gibsoni*	3
*Dirofilaria* spp., *Hepatozoon canis*	2
*Leishmania* spp., *Ehrlichia* spp.	2
*Leishmania* spp., *B. gibsoni*	10
*Ehrlichia* spp., *Anaplasma* spp.	1
*Ehrlichia* spp., *H. canis*	1
*Angiostrongylus vasorum*, *Dirofilaria* spp.	1
*Dirofilaria* spp., *Ehrlichia* spp., *Leishmania* spp.	1
*Dirofilaria* spp., *Anaplasma* spp., *B. gibsoni*	1
*Dirofilaria* spp., *Leishmania* spp., *B. gibsoni*	1
Total	26

In the risk factor analysis, none of the tested independent variables were associated with tick-borne infection (Table 5); therefore, multivariable modeling was not performed.

**Table 5:** Univariable associations between tick-borne infection status and the independent variables in 158 study dogs using simple logistic regression

Category	No. positive (%)	No. negative (%)	Odds ratio (95% CI)	Statistical analysis
Sex				
Male	12 (22.6)	41 (77.4)	–	*χ*^2^ = 1.66, *df* = 1, *P* = 0.205
Female	34 (32.4)	71 (67.6)	1.64 (0.8–3.5)	
Age (years)				
< 1	12 (23.5)	39 (76.5)	–	*χ*^2^ = 1.38, *df* = 2, *P* = 0.501
1 to < 2	20 (32.3)	42 (67.7)	1.55 (0.7–3.6)	
≥ 2	12 (33.3)	24 (66.7)	1.63 (0.6–4.2)	
Outdoor walks (per day)				
< 1	5 (19.2)	21 (80.8)	–	*χ*^2^ = 5.08, *df* = 3, *P* = 0.166
1	7 (19.4)	29 (80.6)	1.01 (0.3–3.6)	
2	10 (32.3)	21 (67.7)	1.99 (0.6–6.9)	
≥ 3	24 (37)	41 (63)	2.46 (0.8–7.4)	
Stray				
Yes	29 (30.8)	65 (69.2)	–	*χ*^2^ = 0.34, *df* = 1, *P* = 0.561
No	17 (26.6)	47 (73.4)	1.23 (0.6–2.5)	
Environment (access to vegetation)				
Limited	8 (28.6)	20 (71.4)	–	*χ*^2^ = 0.001, *df* = 1, *P* = 0.944
Yes	38 (29.2)	92 (70.8)	1.03 (0.4–2.6)	

χ^2^ Chi-square value, *df* degrees of freedom, CI confidence interval

## Discussion

Almost half of the dogs in this study tested positive for at least one VBP, suggesting that CVBDs are highly prevalent among owned and stray dogs with outdoor access in Hong Kong. Notably, 11.4% of dogs were seropositive for *Leishmania* spp., yet no *Leishmania* DNA was detected in any blood sample. In naturally occurring *Leishmania* spp. infections, due to their tropism for lymphoid tissues, protozoa are more abundant in lymph node and bone marrow aspirates, as well as in conjunctival swab samples, than in whole blood [23]. However, as none of these samples were collected, *Leishmania* infection could not be confirmed.

Although no competent sand fly vectors have yet been reported in Hong Kong, *Phlebotomus chinensis*, a known vector of visceral leishmaniasis in China, has been detected in the nearby provinces of Hainan and Guangdong in mainland China, suggesting that an expansion of the geographical distribution of these vectors may have occurred [24–26]. In 2023, the first case of human visceral leishmaniasis in nearby Shenzhen city (Guangdong province) was reported, although it was thought to be an imported case from an endemic area in China [27]. Sand flies belonging to the genus *Sergentomyia*, which prefer to feed on cold-blooded vertebrates, have been found in Hong Kong [24] and are widely distributed in the Old World [28]. The role of the herpetophilic *Sergentomyia* in the transmission of zoonotic *Leishmania* spp. is controversial, as *L. infantum* [29–31] and *Leishmania martiniquensis* [32] have been molecularly detected in these sand flies.

In the present study, samples were tested for antibodies to *Leishmania* spp. using a POC immunoassay and IFAT, with the latter being considered the reference test among serological tests [33]. The lack of agreement between the POC immunoassay and IFAT could be due to the high sensitivity of IFAT for the detection of subclinically infected dogs [19, 34, 35]. Low IFAT titers, which were most common in our study, are difficult to interpret, especially in the absence of molecular confirmation of the infection. Although infections by *T. evansi* were ruled out by PCR, the IFAT seropositivity obtained is unlikely to be due to cross-reactivity with trypanosomatids [35, 36]. Nonetheless, it should be acknowledged that in China, other *Leishmania* spp. (e.g., *Leishmania gerbilli*, *Leishmania tropica*, *Leishmania turanica*, and an undescribed *Leishmania* sp. closely related to *Sauroleishmania*) have been identified [37], including in dogs [38]. Previous studies have suggested that cross-reactivity between *Leishmania* spp. and *E. canis* may occur [39–41]. In our study, three of the 17 *Leishmania*-seropositive dogs were infected with *E. canis*, while overall, 15 (88.2%) of *Leishmania*-seropositive samples were co-infected with VBPs (Table 4); thus, the occurrence of cross-reactivity with other VBPs cannot be ruled out.

The proportion of dogs testing positive for *D. immitis* antigens by POC assay (19%; 95% CI 12.9–25.1) is higher than that previously recorded in Hong Kong (i.e., 6% and 5.6%) [6, 14], suggesting an increasing distribution of this mosquito-borne infection and the importance of adopting preventive chemoprophylaxis control measures in dog populations. While the prevalence in this study was similar among stray (19.1%) and owned dogs (23.4%), most infected animals (69.7%) were older than 1 year, reflecting increased opportunities for vector exposure when compared to younger animals. The finding of dogs PCR-positive for *D. immitis* but antigen-negative by POC assays may be due to a low burden of adult female worms [42–44]. However, as no heat pretreatment of sera was performed before the use of the POC assay, immune complex formation resulting in antigen-negative results on the POC assay cannot be excluded [45]. Conversely, the antigen positivity detected in a sample that was molecularly positive for *D. asiatica* might suggest a simultaneous infection with *D. immitis,* or might be due to a cross-reaction between the two *Dirofilaria* spp.

Although *W. pipientis* DNA can originate from several filarioids (e.g., *Acanthocheilonema* spp., *Dirofilaria* spp., *Brugia* spp.) [46], we only detected DNA of *Dirofilaria* spp., supporting *Wolbachia* spp. as an indicative marker for *D. immitis* infection in this study [47, 48].

The finding of one healthy dog seropositive for *A. vasorum* is of interest, since it has not been previously detected in East or Southeast Asia, and infected dogs usually present with severe respiratory signs [49]. Although *A. vasorum* has been detected in Turkey, which lies mainly in West Asia, its presence in other parts of Asia has not been documented [50]. However, as other *Angiostrongylus* spp., such as *A. cantonensis* and *A. malaysiensis*, have been reported in East and Southeast Asia [51, 52], cross-reactivity [53] might have occurred. The *A. vasorum*-seropositive dog in this study also tested positive for *D. immitis* on both POC immunoassays, as well as by PCR. However, cross-reactivity with *A. vasorum* on the Angio Detect™ Test is highly unlikely, as this event was excluded in purpose-designed studies [20, 54]. Accordingly, the *A. vasorum* antigen assay employed was reported to have an overall sensitivity of 85% (combined for experimentally infected dogs and field infections), and a specificity of 100% [20, 55]. Other investigators found cross-reactivity of the *A. vasorum* POC assay used in this study with other *Angiostrongylus* spp. infecting wildlife in Europe [56] or with a close relative from the family Angiostrongylidae, *Gurltia paralysans,* in cats from Chile [57]. Thus, further research is needed to confirm the presence of *A. vasorum* in Hong Kong (e.g., Baermann sedimentation with molecular confirmation).

Overall, nearly one-third (29.1%) of the dogs in this study were infected with at least one tick-borne pathogen, indirectly illustrating the tick abundance in Hong Kong, with *R. sanguineus* s.l. and* H. longicornis* being common in East Asia [58–60]. The employment of PCR protocols amplifying more than one pathogen, such as in the case of piroplasms and rickettsias, represents a limitation of this study, as it may have underestimated the occurrence of co-infections.

The higher prevalence of *A. platys* than *E. canis* detected in this study (i.e., 7.6% vs. 3.8%) differs from previous studies conducted on owned dogs in Hong Kong, which found a mean of 10.8% for *Ehrlichia* versus 2.7% for *Anaplasma* [6, 14], and might be related to the sampled population of dogs. Specifically, previous studies focused predominantly on sick dogs for which veterinary care was sought, compared to the healthy dog population in this study. Infection by *E. canis* often presents as a severe clinical illness, in contrast to *A. platys* infections, which are typically subclinical [61].

The discrepancies between PCR and serology results for *Ehrlichia* and *Anaplasma* are likely related to the stage of infection, since PCR-positive results are expected in acute-stage infection [62] while seropositivity may reflect past or chronic exposure [63]. Similar to other investigations in Hong Kong [6, 13, 14], *E. canis* was the only *Ehrlichia* sp. detected in our study, reflecting the distribution of *R. sanguineus* s.l. vectors [12].

The results of this study suggest that after dirofilariosis, babesiosis caused by *B. gibsoni* is the second most common CVBD in Hong Kong, with a decreasing prevalence in the last 20 years from 41% overall in stray and owned dogs [14] to 15.2% in the present study.

## Conclusions

Overall, this study highlights the circulation of several zoonotic VBPs in dogs from Hong Kong, with *D. immitis* being the most common among both owned and stray animals. In addition, *Leishmania* spp. seropositivity was detected in dogs, highlighting the need for prospective studies to confirm *Leishmania* infection and to perform surveillance for competent vectors. Given the high exposure to arthropod-borne pathogens, year-round parasiticide treatment of animals in combination with insecticides and repellents is advocated to mitigate the risk of CVBD in dogs and in humans.

## Supplementary Information

Supplementary Material 1.

## Data Availability

Sequences generated in this study are available in the GenBank database (i.e., *Dirofilaria immitis:* PV636295-6310; *Dirofilaria* sp. “hongkongensis”: PV636311-2; *Babesia gibsoni*: PV636262-84; *Hepatozoon canis*: PV636285-91; Uncultured *Anaplasma* sp.: PV636148-51; Uncultured *Ehrlichia* sp.: PV636152-3).

## Abbreviations

PCR: Polymerase chain reaction
qPCR: Quantitative polymerase chain reaction
CI: Confidence interval
IFAT: Immunofluorescence antibody test
ELISA: Enzyme-linked immunosorbent assay
kDNA: Kinetoplast DNA
IgG: Immunoglobulin G
