# Supplementary material for: High prevalence of vector-borne pathogens in the blood of clinically healthy dogs in Hong Kong
Source: Parasit Vectors. 2025 Jul 20;18:289. doi: 10.1186/s13071-025-06853-5 (PMC12278506; doi:10.1186/s13071-025-06853-5)
Supplement: Supplementary file 1 — Supplementary Material 1. [file 13071_2025_6853_MOESM1_ESM.docx]

**Supplementary table 1**. NCBI GenBank BLAST results for sequenced PCR positive samples for canine vector-borne pathogens in Hong Kong.

| **Sample Identification number** | **Species (BLAST)** | **GenBank Accession number** | **Geographical location** | **Host and source** | **Amplicon size** | **Query cover (%)** | **ID %** |
| --- | --- | --- | --- | --- | --- | --- | --- |
| 3 | *Dirofilaria immitis* | MW577348 | Thailand | Human eye | 669 | 100 | 100 |
| 8 | *Dirofilaria immitis* | MW577348 | Thailand | Human eye | 664 | 100 | 100 |
| 19 | *Dirofilaria immitis* | MW577348 | Thailand | Human eye | 674 | 100 | 100 |
| 41 | *Dirofilaria immitis* | MW577348 | Thailand | Human eye | 660 | 100 | 100 |
| 46 | *Dirofilaria immitis* | MW577348 | Thailand | Human eye | 670 | 100 | 100 |
| 47 | *Dirofilaria immitis* | MW577348 | Thailand | Human eye | 664 | 100 | 100 |
| 60 | *Dirofilaria immitis* | MW577348 | Thailand | Human eye | 674 | 100 | 100 |
| 78 | *Dirofilaria immitis* | MW577348 | Thailand | Human eye | 668 | 100 | 100 |
| 92 | *Dirofilaria immitis* | MW577348 | Thailand | Human eye | 663 | 100 | 100 |
| 111 | *Dirofilaria immitis* | MW577348 | Thailand | Human eye | 665 | 100 | 100 |
| 112 | *Dirofilaria immitis* | MW577348 | Thailand | Human eye | 664 | 100 | 100 |
| 124 | *Dirofilaria immitis* | MW577348 | Thailand | Human eye | 668 | 100 | 100 |
| 134 | *Dirofilaria immitis* | MW577348 | Thailand | Human eye | 640 | 100 | 100 |
| 135 | *Dirofilaria immitis* | MW577348 | Thailand | Human eye | 624 | 100 | 100 |
| 136 | *Dirofilaria immitis* | MW577348 | Thailand | Human eye | 665 | 100 | 100 |
| 137 | *Dirofilaria immitis* | MW577348 | Thailand | Human eye | 664 | 100 | 100 |
| 28 | *Dirofilaria* sp. “hongkongensis” | NC_031365 | India | Human eyelid | 672 | 100 | 100 |
| 147 | *Dirofilaria* sp. “hongkongensis” | NC_031365 | India | Human eyelid | 640 | 100 | 100 |
| 13 | *Anaplasma platys* | MG050139 | India | *Rhipicephalus sanguineus* | 256 | 100 | 100 |
| 14 | *Anaplasma platys* | MG050139 | India | *Rhipicephalus sanguineus* | 265 | 100 | 100 |
| 32 | *Anaplasma platys* | MG050139 | India | *Rhipicephalus sanguineus* | 263 | 100 | 100 |
| 141 | *Anaplasma platys* | EF139459 | Thailand | Canine blood | 263 | 100 | 100 |
| 21 | *Ehrlichia canis* | PP321265 | Bangladesh | Canine | 261 | 100 | 100 |
| 94 | *Ehrlichia canis* | OP164592 | Thailand | Canine | 257 | 100 | 100 |
| 53 | *Ehrlichia canis* | PP321265 | Bangladesh | Canine | 261 | 100 | 100 |
| 4 | *Babesia gibsoni* | MN134516 | India | Canine | 479 | 100 | 100 |
| 7 | *Babesia gibsoni* | MN134516 | India | Canine | 481 | 100 | 100 |
| 19 | *Babesia gibsoni* | MN134516 | India | Canine | 346 | 100 | 98.55 |
| 22 | *Babesia gibsoni* | MN134516 | India | Canine blood | 466 | 100 | 100 |
| 24 | *Babesia gibsoni* | MN134516 | India | Canine blood | 430 | 100 | 100 |
| 42 | *Babesia gibsoni* | MN134516 | India | Canine blood | 426 | 100 | 100 |
| 76 | *Babesia gibsoni* | MN134516 | India | Canine blood | 473 | 100 | 100 |
| 77 | *Babesia gibsoni* | MN134516 | India | Canine blood | 479 | 100 | 100 |
| 81 | *Babesia gibsoni* | MN134516 | India | Canine blood | 468 | 100 | 100 |
| 82 | *Babesia gibsoni* | MN134516 | India | Canine blood | 468 | 100 | 100 |
| 83 | *Babesia gibsoni* | MN134516 | India | Canine blood | 459 | 100 | 100 |
| 84 | *Babesia gibsoni* | MN134516 | India | Canine blood | 443 | 100 | 100 |
| 87 | *Babesia gibsoni* | MN134516 | India | Canine blood | 469 | 100 | 100 |
| 92 | *Babesia gibsoni* | MN134516 | India | Canine blood | 477 | 100 | 100 |
| 97 | *Babesia gibsoni* | MN134516 | India | Canine blood | 414 | 100 | 100 |
| 105 | *Babesia gibsoni* | MN134516 | India | Canine blood | 317 | 100 | 100 |
| 119 | *Babesia gibsoni* | MN134516 | India | Canine blood | 477 | 100 | 100 |
| 123 | *Babesia gibsoni* | MN134516 | India | Canine blood | 464 | 100 | 100 |
| 127 | *Babesia gibsoni* | MN134516 | India | Canine blood | 449 | 100 | 100 |
| 133 | *Babesia gibsoni* | MN134516 | India | Canine blood | 474 | 100 | 100 |
| 142 | *Babesia gibsoni* | MN134516 | India | Canine blood | 476 | 100 | 100 |
| 149 | *Babesia gibsoni* | MN134516 | India | Canine blood | 458 | 100 | 100 |
| 152 | *Babesia gibsoni* | MN134516 | India | Canine blood | 478 | 100 | 100 |
| 154 | *Babesia gibsoni* | MN134516 | India | Canine blood | 414 | 100 | 100 |
| 9 | *Hepatozoon canis* | LC556379 | Malawi | Canine blood | 521 | 100 | 100 |
| 23 | *Hepatozoon canis* | PV077335 | Italy | Canine blood | 541 | 100 | 100 |
| 28 | *Hepatozoon canis* | PV077335 | Italy | Canine blood | 543 | 100 | 100 |
| 29 | *Hepatozoon canis* | PV077335 | Italy | Canine blood | 540 | 100 | 100 |
| 46 | *Hepatozoon canis* | PV077335 | Italy | Canine blood | 540 | 100 | 99.81 |
| 53 | *Hepatozoon canis* | PV077338 | Italy | Canine blood | 535 | 100 | 99.81 |
| 69 | *Hepatozoon canis* | LC556379 | Malawi | Canine blood | 499 | 100 | 99.80 |
